# A tool for predicting pH and temperature effects on porcine and human pepsin activity during *in vitro* gastric digestion

**DOI:** 10.1038/s41598-026-38033-5

**Published:** 2026-02-15

**Authors:** Fitzpatrick C. J., Freitas D., Comi I., Vegarud G. E., Røseth A. G., Hayes E., O’Callaghan T. F., O’Mahony J. A., Brodkorb A.

**Affiliations:** 1 Teagasc, Moorepark Research Centre, Fermoy, Co., Cork, P61 C996 Ireland; 2 School of Food and Nutritional Sciences, University College Cork, Cork, T12 K8AF Ireland; 3 Vistamilk RI Research Centre, Teagasc, Moorepark, Fermoy, Co., Cork, P61 C996 Ireland; 4 Faculty of Chemistry, Biotechnology and Food Science, Norwegian University of Life Sciences, Aas, 1433 Norway; 5 Department of Internal Medicine, Lovisenberg Diaconal Hospital, Oslo, 0456 Norway

**Keywords:** Porcine pepsin, Human pepsin, Prediction model, INFOGEST, Digestion, Denaturation, Biochemistry, Proteases

## Abstract

*In vitro* food digestion methods are increasingly used due to standardised protocols and validation with *in vivo* data. Porcine pepsin is the major gastric enzyme used to replace human pepsin during the gastric phase of *in vitro* digestion models. Until now, limited information exists on the combined effect of the temperature and pH variations that occur during digestion on the activity of these enzymes. This study addresses gaps in the understanding of how pH (1–7) and temperature (4–60 °C) affect pepsin activity. Human pepsin exhibited broader activity retention than porcine pepsin, maintaining 80% and 46% activity at pH 3 and 4, respectively, versus 47% and 13% for porcine pepsin. Predictive models for both enzymes were developed to quantify activity under varied conditions. Porcine pepsin required heating to 75 °C for ≥ 5 min for complete inactivation, while 65 °C for 15 min proved insufficient. These findings allow direct comparison of standardised* in vitro* digestion results across studies, improving the translation of *in vitro* outcomes to *in vivo* human digestion. Additionally, this study clarifies optimal inactivation protocols for sample preparation. This work therefore enhances the accuracy of in vitro models, supporting their use in food research.

**Supplementary Information:**

The online version contains supplementary material available at 10.1038/s41598-026-38033-5.

## Introduction

Pepsin (EC 3.4.23.1) is an endopeptidase that plays a critical role in digestion. This enzyme is secreted in an inactive form (pepsinogen) by gastric chief cells and is activated on reaching the acidic environment of the stomach. As with all enzymes, its activity can be strongly affected by pH and temperature. As a result, changes in these parameters within the gastric compartment can influence the rate, pattern and extent of protein digestion.

The activity of pepsin in human gastric fluid (hereinafter referred to as human pepsin) has been shown to remain stable at pH up to 6; however, while stable, very little activity remains at pH > 5.5, with optimum activity at pH 2 at 37 °C^1–5^. Gastric pH is strongly affected by the pH and buffering capacity of the meal consumed, as well as the dynamic aspects of gastric digestion (secretion of hydrochloric acid from parietal cells). Several studies have shown the large fluctuations in gastric pH, as described in Table 1.

**Table 1:** Summary of gastric pH observations in humans in response to different meal types and compositions.

Meal description	Gastric pH observations	Time to pH 2	References
Hamburger, bread, hash brown potatoes, vegetables, milk (~ 600 g total weight and 1,000 kcal)	Fasted state pH: 1.7; increased to 4.3–5.4 (peak: 6.7) during ingestion; decreased to pH 2.0 gradually.	107 ± 70 min	^2^
Lean steak, boiled potatoes, vegetables, salad (575–675 g); dessert (200 mL), water (200 mL). Meal pH 5.9	Gastric pH increased to 4.5 after meal ingestion; returned to pH 2.0 more gradually.	Average of 240 min	^3^
Liquid meal: 500 mL (376.5 kcal: 14 g protein, 52 g carbohydrate, 12.5 g lipid); meal pH: 7.	Initial pH: ~7 (similar to meal pH); decreased gradually as the stomach emptied; reached pH 2 when 90% of the meal was emptied.	> 150 min	^4^

Studies have also shown that a large variation in gastric temperature can occur due to the ingestion of a meal. When 400 mL of orange juice was consumed at 50 °C, it was found that gastric temperatures increased to 43 °C, while consuming the same beverage at 4 °C caused gastric temperatures to reduce to 21 °C^5^. Gastric temperature then returned to within 1 °C of body temperature within 20 min of ingestion of the warm drink and within 30 min of ingestion of the cold drink. McArthur and Feldman^5^ also investigated gastric temperature fluctuations using 360 mL of coffee (infused into the stomach) as a test drink. They found that when coffee was consumed at 4–58 °C, it took 16.7 ± 2.7 min and 23.8 ± 1.1 min to return to 37 °C^5^.

These findings demonstrate the large variations that can be observed in both gastric pH and temperature in the postprandial period and, since pepsin activity is affected by these parameters, suggest possible implications for protein hydrolysis during this digestive step^6^.

Studying digestion in vivo is often difficult; therefore, *in vitro* digestion studies are becoming increasingly common. *In vitro* digestion protocols use simulated digestion fluids and non-human enzymes to mimic *in vivo* digestion. Porcine pepsin is commonly used as a replacement for human pepsin as it exhibits some important similarities with the human counterpart, while being more readily available. Both human and porcine pepsin exhibit similar configurations, displaying 84% structural conservation^7^ and both enzymes have been shown to work with optimum activity at pH close to 2 and temperature close to 37 °C^1,8–10^. However, the effect of concurrent variations in pH and temperature of the food when ingested in each of these enzymes remains poorly understood. As a result, establishing comparisons between the proteolytic capacity of human and porcine pepsins is not straightforward.

As well as this, regardless of the type of *in vitro* digestion used, pepsin activity must be inhibited in digesta samples collected throughout the experiments and after digestion to prevent over estimation of protein breakdown during analysis. Several methods to inhibit enzyme activity exist and should be chosen based on the enzymes present and on the post-digestion analysis to be carried out^11,12^. One method of pepsin inhibition that is commonly used is heat treatment at 100 °C. Indeed, exposure to high temperatures can result in the loss of structural integrity of pepsin, and consequent inactivation. However, this may have repercussions for other components of interest in the samples and possibly impact the outcomes of the study. Therefore, it is best to use the lowest heat treatment possible to minimise the impact on other food compounds, e.g. proteins in the digesta.

Consequently, the main aim of this study was to understand the combined effect of variations in environmental pH and temperature on the activity of human and porcine pepsin and to develop a user-friendly tool that allows users to predict and compare the proteolytic activity of these two enzymes based on the pH and temperature conditions during *in vitro* digestion. Furthermore, this paper aims to investigate the minimum heat treatment required to inhibit pepsin activity in *in vitro* digestion samples.

## Results and discussion

### Pepsin activity at optimum pH and temperature

Human gastric fluid (HGF) pepsin activity was assessed in two laboratories—Teagasc Food Research Centre, Moorepark, Ireland, and the Norwegian University of Biosciences (NMBU), Norway—to confirm that activity was not lost during storage or transport. After centrifugation, HGF was stored at −80 °C for 9 days. Following this, it was transported on dry ice from Norway to Ireland within 48 h where it was again stored at −80 °C.

The activity measured in Ireland was 931.0 ± 110 U/mL, which closely aligned with the measurement in Norway (918 ± 97.7 U/mL). Porcine and human pepsin activities were determined at pH 2 and 37 °C. Porcine pepsin activity was measured at 3,095.2 ± 328.7 U/mg. The values obtained in Ireland were established as the reported optimal (100%) pepsin activities and were used as references to calculate the percentage of pepsin activity at different pH and temperature conditions.

### Production of human and porcine pepsin activity prediction models

**Fig. 1:**
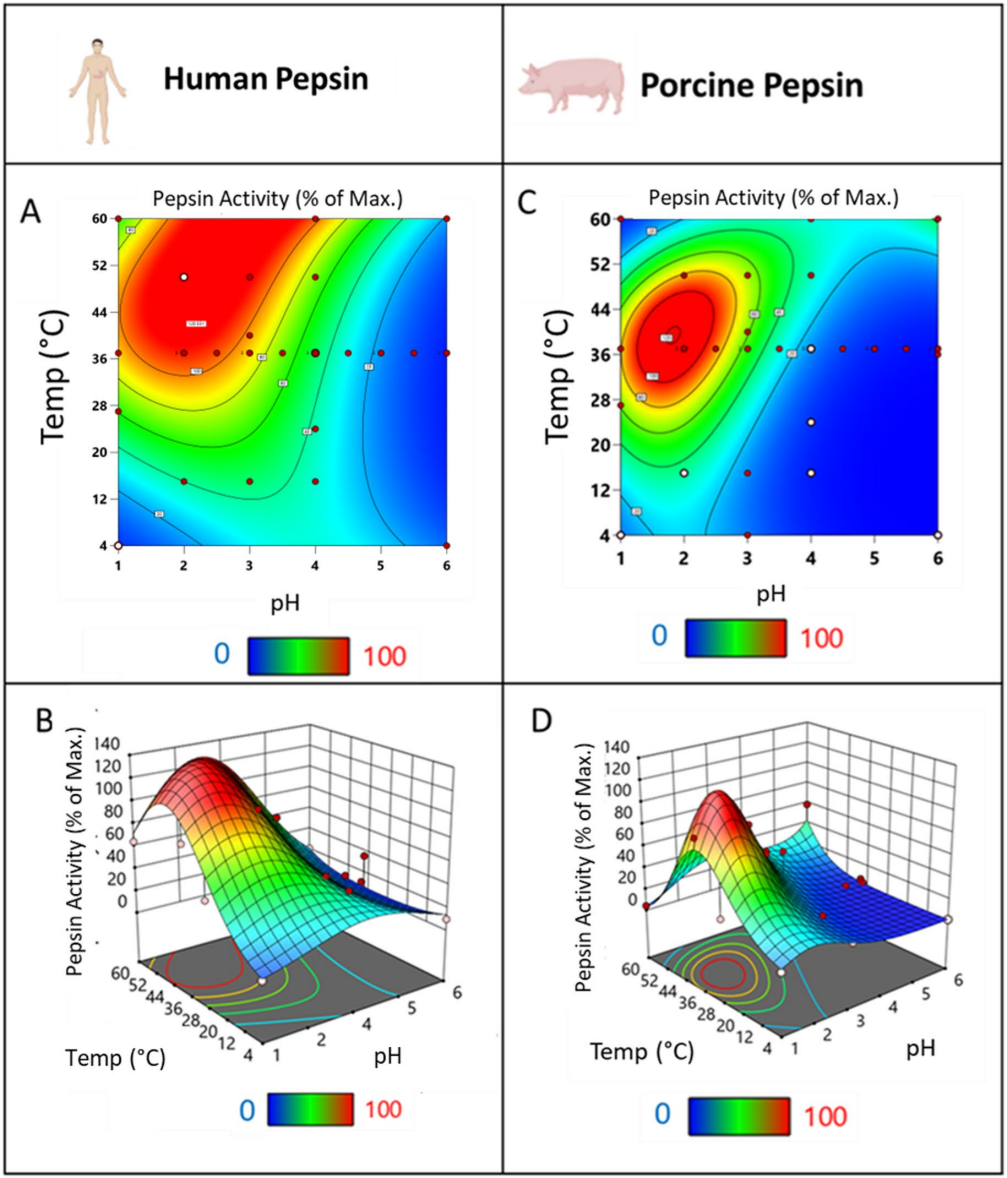
Impact of pH and temperature on pepsin activity. Contour graphs (**A** and **C**) and 3D surface models (**B** and **D**) of pepsin activity in human pepsin (**A** and **B**) and porcine pepsin (**C** and **D**) across a range of pH and temperature (pH 1–7, temp 4–60 °C). Blue areas represent low activity while red areas represent high activity. Pepsin activity is expressed as a percentage of the activity measured under optimum conditions (pH 2 and 37 °C) using haemoglobin as substrate, i.e., 931.0 ± 110 U/mL and 3,095.2 ± 328.7 U/mg for human and porcine pepsin, respectively. One unit is defined as the amount of enzyme that produces a ΔA280 of 0.001 per min at pH 2.0 and 37 °C, measured as TCA-soluble products. Panels B and D show potential activity over 100% due to pepsin activity at certain condition being higher than at pH 2, 37 °C.

Following the activity determinations under optimum pH and temperature conditions, each type of pepsin (human or porcine) was analysed at 37 different pH and temperature combinations (Supplementary Tables 1 and 2, respectively) using a modified version of the INFOGEST pepsin assay (described in Sect. 3.3)^11^. The data collected from these analyses were used to create the human and porcine pepsin activity models. The human pepsin predictive equation is displayed in Eq. 1. From this equation, both contour graphs and 3D surface model graphs were created, highlighting areas of differentiated pepsin activity (Fig. 1A and B). Human pepsin had high activity close to its optimum conditions at pH 2, 37 °C. Its proteolytic activity then decreased as conditions moved away from this pH and temperature, as evidenced by the transition from the red to the blue area of the graph.

$$\begin{aligned}\:\mathrm{log}\left(Pepsin\:Activity\right)=-0.17363+\left(0.76823\mathrm{*}\mathrm{p}\mathrm{H}\right)\\+\left(0.88912\mathrm{*}\mathrm{T}\mathrm{e}\mathrm{m}\mathrm{p}\right)-\left(0.02263\mathrm{*}\mathrm{p}\mathrm{H}\mathrm{*}\mathrm{T}\mathrm{e}\mathrm{m}\mathrm{p}\right)\\-\left(0.08922*p{H}^{2}\right)-\left(0.00107*Tem{p}^{2}\right)+\left(0.00031*pH*Tem{p}^{2}\right)\end{aligned}$$

*Eq. 1: Human Pepsin activity prediction equation. Users can input pH and temperature*,* giving the result as a percentage of optimum activity*.

The porcine pepsin predictive equation is displayed in Eq. 2. From this equation, both contour graphs and 3D surface model graphs were created (Fig. 1C and D*).* Porcine pepsin exhibited high activity close to its optimum conditions at pH 2, 37 °C. However, its activity quickly decreased as conditions moved away from this point, as indicated by the green and blue colours.

$$\begin{aligned}\:log\left(Pepsin\:Activity\right)==-0.104906\:+\left(1.50841\:*\:pH\:\right)+\left(0.03685\:*\:Temp\right)\\\:+(-0.02088\:*\:pH*Temp)\:+(-0.49262\:*\:p{H}^{2})\\+\left(0.00071\:*\:Tem{p}^{2}\right)+\left(0.00045\:*\:pH*Tem{p}^{2}\right)+\left(0.04129\:*\:p{H}^{3}\right)\\+(-0.00003\:*\:Tem{p}^{3}\:)\end{aligned}$$

*Eq. 2: Porcine Pepsin activity prediction equation. Users can input pH and temperature*,* giving the result as a percentage of optimum activity*.

### Comparison between human Pepsin and Porcine Pepsin

When comparing the activity profiles of porcine and human pepsin (Fig. 1), differences in their behaviour across pH and temperature ranges were evident. Human pepsin demonstrated broader regions of high activity, indicated by red areas on the graphs (Fig. 1 (A) and (B)), signifying that it retained greater enzymatic activity over a wider range of temperatures and pH levels compared to porcine pepsin (Fig. 1 (C) and (D)). At pH 2 and 37 °C, human pepsin displayed peak activity. Even as the pH was increased to 3, human pepsin retained over 90% of its activity at 37 °C, with activity still at approximately 38% at pH 4 under the same temperature conditions. In contrast, porcine pepsin exhibited a more rapid decline in activity as pH increased. At pH 2 and 37 °C, porcine pepsin displayed optimum activity, similar to human pepsin. However, at pH 3 and 37 °C, the activity decreased to ~ 57%, decreasing further to below 9% at pH 4. This sharp reduction was more pronounced compared to human pepsin, which still retained considerable activity at higher pH levels. This demonstrated the more restricted range of activity of porcine pepsin, which rapidly lost activity as pH increased beyond 2.50, particularly at physiological temperature (37 °C).

When considering the observed differences in this study, structural differences should be considered. Firstly, porcine pepsin has the amino acid Tyr at position 75, while human pepsin has Phe (Tyr75 > Phe), which may affect the hydrophobic pocket. As well as this, human pepsin contains a Glu-Glu motif within the β-hairpin flap which may affect its optimum pH range^13–15^. Interestingly, porcine pepsin is phosphorylated at Ser68 while human pepsin is not. Moreover, human pepsin exists as multiple isoforms due to post translational modifications (PTMs) such as phosphorylation and glycosylation, which can alter its structure, activity or stability. These isoforms are not reported for porcine pepsin^16^.

Despite the existence of structural differences, processing factors may best explain the broader range of activity observed in human pepsin compared to porcine pepsin. For instance, human pepsin only underwent centrifugation to separate from mucin; as such, it must be considered that it may contain iso-enzymes of pepsin and enzymes other than pepsin, such as gastricsin^9,17^. This may partly explain the broader activity range identified in our study, as gastricsin (Pepsinogen II) has been shown to have an optimum pH at 2.9, and also shows specificity towards the substrate used in the current study (haemoglobin)^18^. In contrast, the porcine pepsin sample was a commercial enzyme preparation, which had been subjected to several purification processing steps. This is likely the major reason for the observed differences in pepsin activity, rather than the minor structural differences that exist between porcine and human pepsin.

The present study, along with previous research, indicates that human pepsin retains its activity over a wider range of conditions compared to porcine pepsin. For example, Benedé, et al^19^. showed that at the start of *in vitro* gastric digestion, β-casein was more rapidly broken down in the presence of human pepsin, compared to porcine pepsin. Moreover, Benedé, et al^19^. also showed that IgE binding sites were more rapidly broken down in B-casein in the presence of human pepsin compared to porcine pepsin.

It should be noted that in the present study, while human pepsin was shown to retain its activity over a wider range of pH and temperature conditions, porcine pepsin displayed higher catalytic activity compared to human pepsin (931.0 ± 110 U/mL in human pepsin vs. 3,095.2 ± 328.7 U/mg in porcine pepsin).

Nevertheless, these findings can have implications for* in vitro* digestion studies as our work suggests that the proteolytic capacity of porcine pepsin under dynamic gastric pH and temperature conditions *in vitro* may differ from what would be expected from human pepsin.

### Case studies

The prediction models created as part of this study for porcine and human pepsin were designed to allow users to quantify pepsin activity throughout *in vitro* gastric model digestion and to compare human vs. porcine pepsin directly. Users can input pH and temperature data from their digestion studies into the Excel-based spreadsheets included in the Supplementary materials. These tools calculate pepsin activity at each data point relative to reported optimal conditions (pH 2, 37 °C), enabling visualisation of activity profiles and area under the curve (AUC) analysis for comparative assessments. Below, case studies are presented to highlight the potential use of these models. It should be noted, however, that the regression model used in this study is based on pepsin activity measured with haemoglobin in solution. Therefore, it does not account for potential interactions between pepsin and the food matrix, which may affect the actual digestion process in complex foods such as milk.

#### Case study 1

**Fig. 2:**
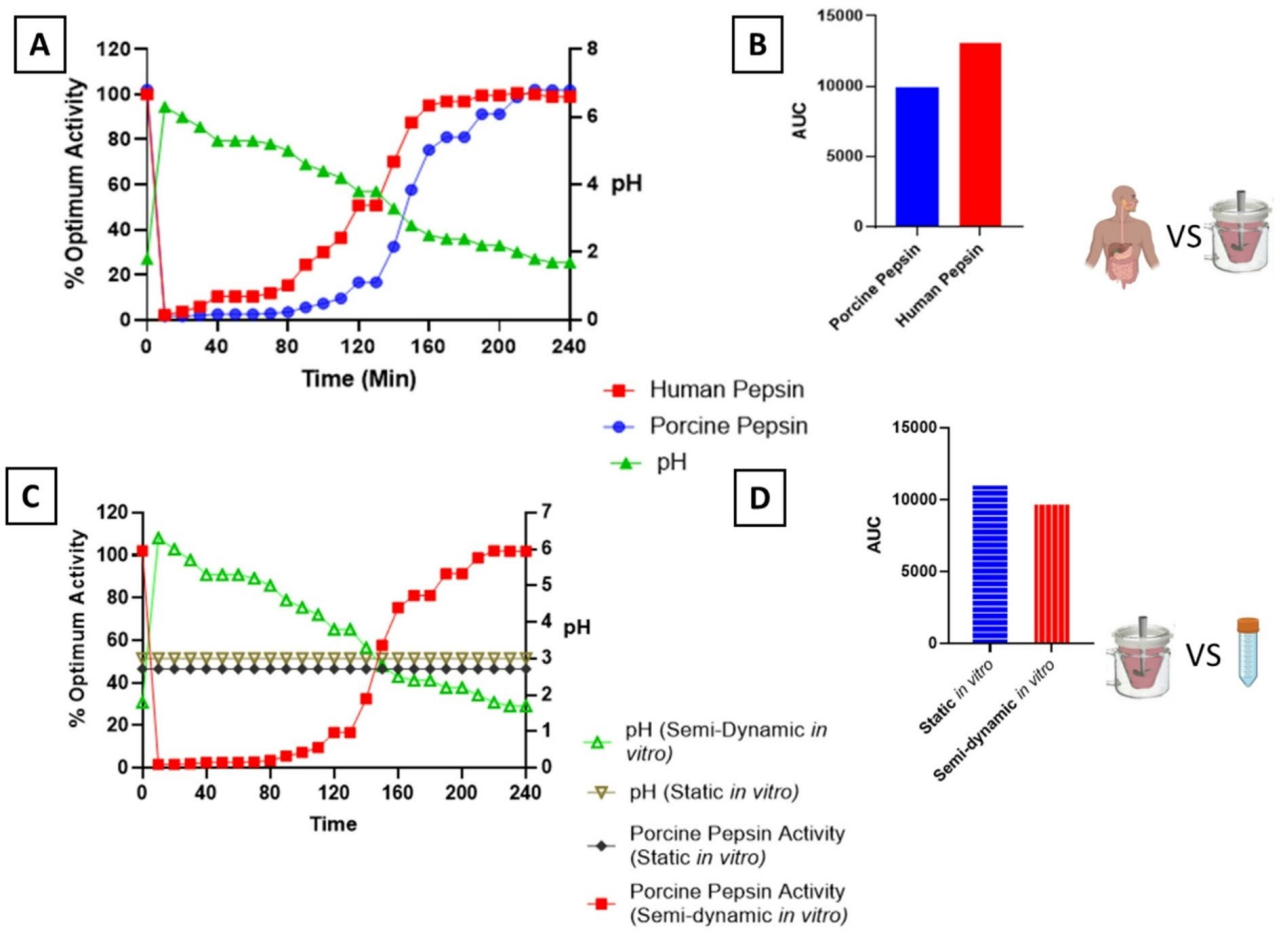
Comparison of different digestion methodologies in terms of pepsin activity. (**A**) Using the same pH curve (Green Triangles), predicted human pepsin activity (Red squares) was compared to predicted porcine pepsin activity over an entire gastric digestion of milk. (**B**) Estimate human pepsin activity was compared to estimated porcine pepsin activity using an area under the curve analysis. (**C**) The predicted porcine pepsin activity was compared in a semi-dynamic *in vitro* digestion (Red squares) to a static *in vitro* digestion (Black Diamonds). Semi-dynamic pH (green triangles) decreased gradually over digestion, while static pH remained constant throughout (Brown upside down triangles). (**D**) Estimate porcine pepsin activity during semi dynamic vs. static *in vitro* digestions were compared.

To provide an example of the use of this model, gastric digestion parameters (temperature, time, and pH) were estimated based on an *in vitro* semi-dynamic gastric digestion of whole milk^15^. Temperature was assumed to be 37 °C for all points in each digestion. This data was then input to both the porcine pepsin model and the human pepsin model; Using both the porcine and human pepsin prediction models, similar curves for human and porcine pepsin during *in vitro* semi-dynamic digestion were observed (Fig. 2, (A)), with pepsin activity being low at the start of digestion and slowly increasing as pH decreases during digestion. However, human pepsin showed a broader range of activity, as illustrated by a greater Area Under the Curve (AUC) value vs. porcine pepsin (Fig. 2 (B)).

#### Case study 2

To test the application of these models for comparisons of the levels of pepsin activity in different* in vitro *digestion models, estimated porcine pepsin activity was compared in static vs. semi-dynamic digestion (Fig. 2 (C)). Static *in vitro* digestion, as expected, showed the same activity throughout digestion due to the maintenance of a constant pH (pH 3), while pepsin activity increased throughout the semi-dynamic digestion. Area Under the Curve calculations (Fig. 2 (D)) were again performed, showing that despite static digestion never reaching the optimum pH of pepsin (pH 2), it had a higher AUC value compared to semi-dynamic digestion over a 240 min gastric digestion. The main reason for this is that in semi-dynamic digestion, users should add HCl to “reach pH 2 at the end of the gastric digestion”^20^. Despite these differences, studies have found a lower release of free amino acids from milk proteins when comparing the static INFOGEST gastric digestion to a dynamic model^19^. This could be due to several confounding factors, such as improved mixing in the dynamic system compared to the static system allowing improved pepsin access to substrate, or a different pH curve in the dynamic model, leading to increased pepsin activity. However, the different substrates in each of these studies are likely one of the main reasons for these differences. Our prediction model would suggest that a higher degree of hydrolysis should be seen when using the static model. In the present experiment, it was shown, using haemoglobin as a substrate that the activity of porcine pepsin was negligible at pH > 4 and increased at pH 3, to an optimum activity at around pH 2. These findings agree with those of Crévieu-Gabriel, Gomez, Caffin and Carré^21^ where haemoglobin was used as a substrate for pepsin activity. In the presence of a different substrate, such as the milk proteins it is possible that pepsin is active at a higher pH^22^. Indeed, changes to the pH range at which porcine pepsin is active with different substrates have been shown elsewhere^23^. Porcine pepsin activity, when measured by the rate of hydrolysis of casein micelle micro aggregates, has been shown to be almost constant in the pH range 1–5^23^. Interestingly, this is consistent with findings from a previous *in vitro* semi-dynamic digestion study, where rapid milk coagulation was observed at the start of the gastric phase (pH ≈ 6)^24^. These findings indicated that pepsin was active at the beginning of digestion, when pH was above the isoelectric point of casein, showing that, although pepsin activity is negligible at high pH when the substance is haemoglobin, activity levels were sufficient to cause coagulation when the substrate was casein.

The capacity of human and porcine pepsin to hydrolyse specific substrates may also be different. As noted by Eriksen, Holm, Jensen, Aaboe, Devold, Jacobsen and Vegarud^9^, pepsin isoforms may interact differently with whey protein compared to haemoglobin. Other studies have also shown that altering the substrate can influence pepsin activity and stability, with Schlamowitz and Peterson^10^ finding that the denaturation of substrates prior to interaction with hog pepsin can increase the pH range that pepsin is active.

#### Case study 3

The pepsin activity prediction model developed in this study can be applied to data to estimate pepsin activity levels during gastric digestion under different conditions. In the present study, adult vs. elderly digestive conditions were compared using porcine pepsin activity model. This model could also easily be replicated with the human pepsin activity model. Several physiological parameters must be considered in this type of analysis, including basal gastric volume, postprandial gastric fluid volume, and pepsin concentration (Table 2). For this simulation, the ingestion of a 50 mL, 50 kcal liquid meal has been considered. By considering gastric fluid secretion rate (0.9 mL/min), rate of pepsin increase (46.8 mg/h) and gastric emptying rate (2 mL/min), the total amount of pepsin in the stomach is calculated at any given time. Pepsin activity is estimated by multiplying the total amount of pepsin by the reported optimal activity value (2,000 U/mg), and the actual activity is predicted using conditions specific to stomach pH and temperature, based on pH data obtained from Gardner, Ciociola and Robinson^3^ and Malagelada, Go and Summerskill^25^. As well as this, the model allows for the simulation of reduced pepsin activity in older adults by implementing a 40% reduction in pepsin output^26^.

**Fig. 3:**
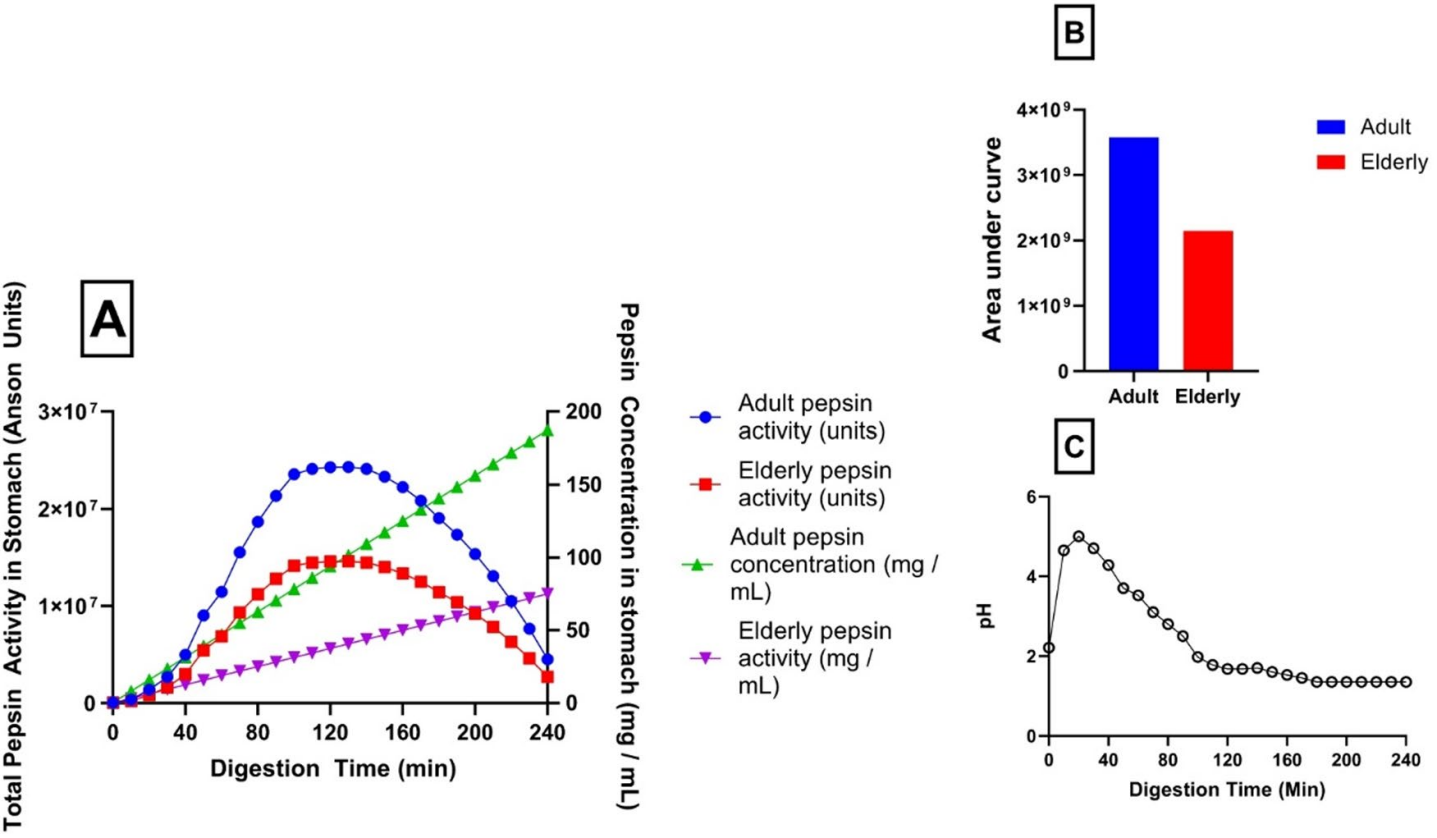
Simulation of total pepsin activity (units) in stomach after the consumption of 50 mL, 50 kCal liquid meal, accounting for basal pepsin concentration, pepsin secretion rate, gastric emptying of pepsin, and pepsin activity based on pepsin activity prediction model. (**A**) Pepsin concentration continues to increase throughout digestion due to continuous pepsin secretion. Pepsin activity increases to approximately 90 min and then begins to decrease due to decreasing total volume of pepsin in stomach due to gastric emptying. (**B** - Inset) Area under the curve calculation for adult vs. older adult digestion. (**C**) pH curve used for this simulation.

Figure 3 (A) shows the predicted change in pepsin activity during digestion of a meal considered here for both adults and older adults *in vivo*. Total pepsin units (main y-axis) are initially low due to the low amount of pepsin present in the stomach, paired with unfavourable conditions for pepsin activity (pH 6) in adults (blue curve). As digestion progressed, an increase in pepsin activity units was observed as more pepsin was secreted into the stomach. At the same time, pH decreased towards pH 2 due to hydrochloric acid secretion, further increasing pepsin activity. After approximately 100 min of digestion, pepsin activity units began to decline as the favourable gastric conditions no longer offset the loss of pepsin due to gastric emptying. A similar trend is seen for older adults (red curve), but with a 40% reduction in pepsin output, resulting in a peak pepsin activity of just over 20,000 units compared to 42,000 units in adults. On the secondary y-axis, pepsin concentration in gastric fluid (mg/mL) is shown for both adults (blue) and older adults (red). Pepsin secretion rate exceeds the loss of pepsin through gastric emptying, leading to a continuous increase in pepsin concentration throughout the digestion period. Over the entire digestion, the area under the curve values for adult total pepsin units is almost twice the value observed for older adults (3.6 × 10^^9^ vs. 2.1 × 10^^9^) for adult vs. older adult, respectively) *(*Fig. 3 (B)).

### Heat deactivation of Pepsin

Heating is a common method of enzyme deactivation used to stabilise samples collected during *in vitro* model digestions. However, if the intensity of heat treatment is too high, it may negatively impact on other proteins of interest in the samples, potentially influencing the outcomes of subsequent analyses. Therefore, best practice is to apply the minimum heat treatment necessary to irreversibly denature pepsin, reducing the risk of undesirable effects on other labile components.

**Fig. 4:**
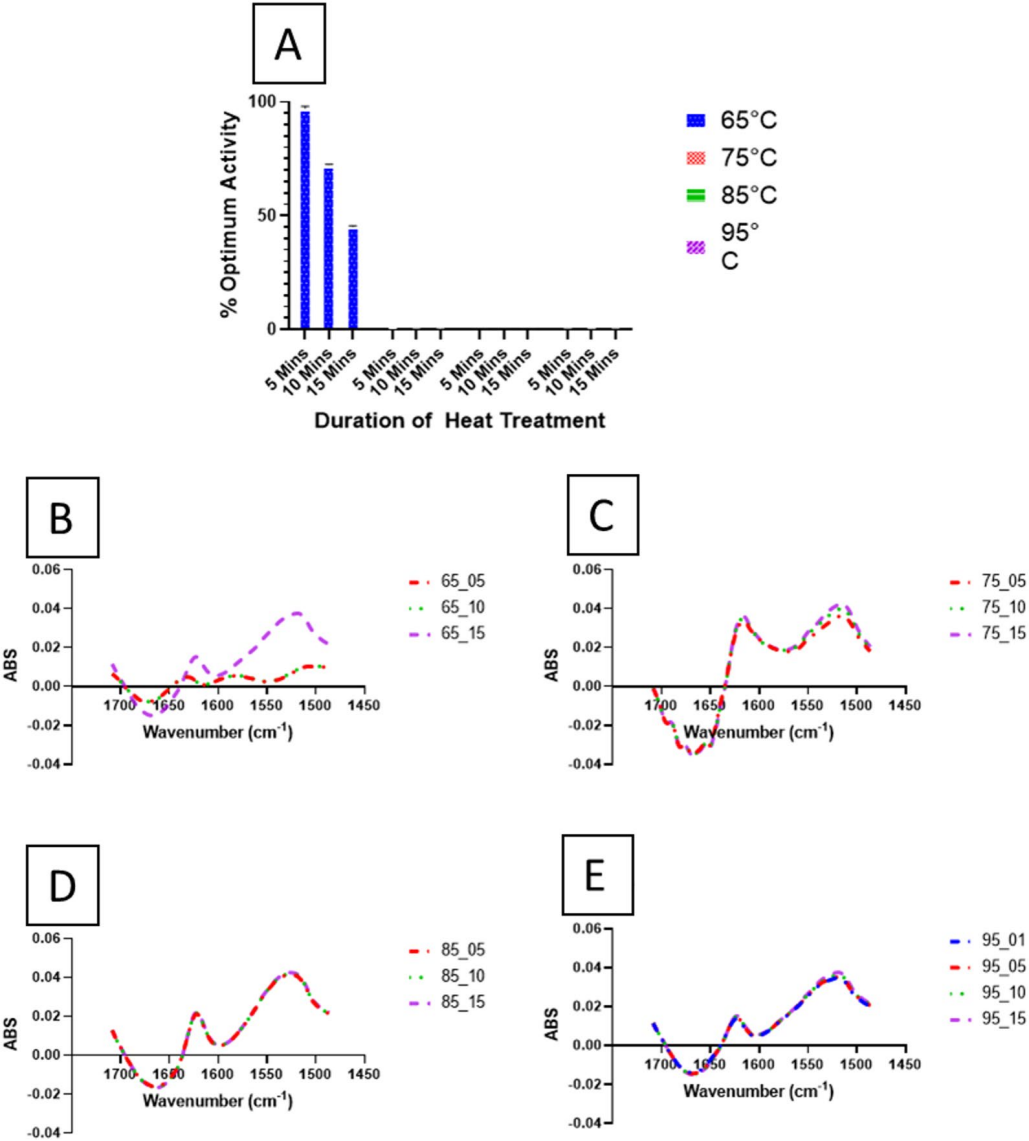
Thermal inactivation of porcine pepsin and corresponding FTIR spectral analysis. (**A**) Pepsin activity was measured at 37 °C (pH 2, using haemoglobin as substrate) following heat treatment at 65 °C, 75 °C, 85 °C, and 90 °C for 5, 10, and 15 min. Activity was preserved after heating at 65 °C across all time points, while any exposure above 75 °C for 5 min resulted in irreversible inactivation (hence, no bars are visible at T > 75 °C). (**B**) FTIR spectra of pepsin under various thermal conditions with native pepsin spectra subtracted: (**B**) 65 °C, (**C**) 75 °C, (**D**) 85 °C, and (**E**) 95 °C. The native pepsin spectrum is provided in supplementary materials (Sup. Figure 1).

In the present study, porcine pepsin was heated to between 65 and 95 °C for set time periods (5, 10–15 min), and activity was subsequently measured at 37 °C. The measured activity was compared to the previously determined ‘optimum’ activity and expressed as a percentage of that value, as shown in Fig. 4A. Heating for 15 min at 65 °C was insufficient to fully deactivate pepsin; after 5 min of heating at 65 °C, pepsin retained 95.35 ± 0.75% of its activity. Extending the heating duration to 10–15 min led to a significant reduction in activity, but 72.37 ± 0.26% and 45.21 ± 0.43% of activity was still retained, respectively. A significant difference (*p <* 0.01) was found at 65 °C between 5 and 10 min *(p <* 0.01), 5 and 15 min (*p <* 0.01), and 10 and 15 min (*p <* 0.01). Heating at higher temperatures led to faster and complete inactivation of pepsin, with 5 min at 75 °C being sufficient to achieve irreversible inactivation. All heat treatments tested at 75 °C, and above, were effective in irreversibly inactivating pepsin and no significant difference was found between heating times. When comparing temperatures, significant differences were found between 65 °C and all other temperatures.

To further investigate the structural changes induced by the different heat treatments and the reversibility of pepsin denaturation after heat treatment, FTIR analysis was carried out on porcine pepsin samples subjected to the same heat treatments (heating to 65–95 °C, over 5–15 min). The results are presented in Fig. 4, showing FTIR spectra for samples heated at 65 °C (B), 75 °C (C), 85 °C (D), and 95 °C (E). The amide I and amide II regions, (1,700-1,600 cm^−1^ and 1,570-1,500 cm^−1^, respectively) were analysed to examine changes in pepsin structure upon heating. The region 1,640-1,630 cm^−1^ is associated with β-sheet structural changes in proteins^27^. A peak shift from 1,636 to 1,626 cm^−1^ was observed after 15 min of heating at 65 °C (Fig. 4 (B), and at all-time points, in all measured temperatures greater than 65 °C (Fig. 4 (C-E), indicating a tighter packing of β-sheets, possibly due to aggregation, which occurs when protein is denatured. When temperature was increased from 65 to 75 °C and higher, an increase in absorbance in this amide I region was seen, which could be related to increased intermolecular β-sheet structures, and an increase in protein unfolding^28^.

The *amide II* region is mainly associated with N-H bending and C-N stretching vibrations of molecules. Changes in absorbance in this region can be related to changes in the α-helical structure of proteins, or conformational changes in proteins. There was an increase in absorbance in the *amide II* region with heating at 65 °C for 15 min (compared to the same temperature and shorter periods), likely resulting from unfolding of the secondary structures. These absorbance levels were comparable to those observed for all heating periods at temperatures of 75, 85 and 95 °C. At these temperatures, pepsin was rapidly denatured with no activity detected (at 37 °C), regardless of the duration (Fig. 4A). Interestingly, nearly overlapping spectra were observed for samples heated 5 and 15 min at each of these higher temperatures. These findings, when considered with the fact that pepsin activity is not recovered after heating to 75 °C and above, suggests that the conformational changes to pepsin are irreversible after heating to this temperature. Conversely, the activity of pepsin after heating at 65 °C, even after 15 min, is partly retained (upon cooling to 37 °C). This finding can be explained by the FTIR data, where no peak shift can be seen at the amide 1 region when pepsin is heated to 65 °C, compared to clear changes in this region at all other tested temperatures.

## Materials and methods

### Materials

Bovine haemoglobin (SBV9700, Sigma-Aldrich^®^) was purchased from Merck (Co. Wicklow, Ireland). Haemoglobin was dissolved 2% (w/v) in water. Porcine pepsin (SLBZ 7282, Sigma-Aldrich^®^), also purchased from Merck (Co. Wicklow, Ireland), was dissolved to 1 mg/mL in 10 mM Tris Buffer with 150 mM NaCl at pH 6.5. Human gastric fluid pepsin was collected which contained pepsin (Refereed to in this paper as human pepsin) from a total of 10 fasting volunteers at Lovisenberg Diaconal Hospital, Oslo. GI fluids were collected, treated and further tested as detailed described according to Asledottir, Comi, Devold, Røseth, Valeur and Vegarud^29^.

### Ethical approval

The aspiration of gastric fluid and its subsequent storage were conducted in accordance with ethical guidelines and approved by the Norwegian Ethics Committee. The study protocol for gastric fluid aspiration was approved under ethics license number REK 2012/2030. Additionally, the storage of human aspirates in the Biobank was approved under ethics license number REK 2012/2010. All volunteers provided written informed consent prior to participating in the study.

### Pepsin activity determinations

When pepsin is assayed using the method outlined in the standardised INFOGEST protocol, it is recommended to use bovine haemoglobin as a substrate^11,30^. During this assay, pepsin is incubated with haemoglobin, releasing tyrosine-containing peptides soluble in trichloroacetic acid (TCA) that can be detected spectrophotometrically (at 280 nm) . Unit definition is as follows “One unit will produce a ΔA_280_ of 0.001 per min at pH 2.0 and 37 °C, measured as TCA-soluble products”^11^. In the present study, both porcine and human samples were firstly assayed in triplicate, using this method (at pH 2, 37 °C) and the resulting values were taken as the respective maximum activities. A modified version of the assay was then used to analyse each sample at a range of pH and temperature conditions (pH 1–7, temp 4–60 °C) as described below.

#### Activity of human pepsin under optimum pH and temperature

A 50 µL/mL solution of human pepsin in 10 mM HCl was prepared. Haemoglobin solution was adjusted to pH 2 and heated to 37 °C. An aliquot (100 µL) of the human pepsin solution in 10 mM HCl was added to 500 µL of haemoglobin and incubated for 10 min at 37 °C. Following this, 1 mL of TCA (5% w/v) was added to stop the reaction. Samples were centrifuged (6,000 × g, 4 °C, 30 min) using a Thermo Scientific Heraeus Multifuge X1R (Thermo Fisher Scientific, USA) centrifuge and supernatant was removed to fresh tubes. Absorbance of supernatant was read at 280 nm.

#### Activity of Porcine Pepsin under optimum pH and temperature

The same procedure was followed, starting from a 1 mg/mL porcine pepsin solution which was diluted to 20 µg/mL in 10 mM HCl and used in place of the human pepsin.

#### Effect of pH and temperature on human and Porcine Pepsin activity

A response surface methodology (RSM) approach was used to systematically select pH and temperature combinations relevant to gastric digestion^30^. A D-optimal design was generated using Design Expert 12 (Design-Expert^®^ version 12.0.9.0, Stat-Ease Inc., USA), selecting 26 combinations of pH and temperatures relevant to gastric digestion to ensure a balanced and orthogonal experimental design. Additionally, 11 extra points were included to optimise the model for semi-dynamic digestions. The impact of these 11 pH and temperature combinations on porcine pepsin had been previously studied in our laboratory and presented with results of an* in vitro* digestion study by Freitas, Gómez-Mascaraque and Brodkorb^31^. Human and porcine samples were therefore assayed at a total of 37 different temperature and pH combinations (presented in Supplementary Tables 1 and 2, respectively). The pH and temperature of the haemoglobin solution (500 µL) was adjusted to the required conditions prior to the addition of human or porcine sample solutions (100 µL) prepared as described above. The incubation was then followed at the required temperature prior to the TCA precipitation and spectrophotometric determination steps carried out according to the recommended protocol described above^11^. Enzyme activity was expressed as proportion (%) of optimum activity, calculated by dividing obtained absolute activity by the activity of the same enzyme at pH 2, 37 °C. When not in use, stock and sample solutions were stored on ice.

### Development of predictive Pepsin activity models

Using the pepsin activity data described in the previous section, two pepsin activity prediction models were developed using Design Expert for porcine and human pepsin activity. For porcine pepsin, a base 10 log transformation was used on the data due to a high maximum to minimum value ratio (114.3 to 0.54 = ratio of 212.80). Terms included in the model were the intercept, A (pH), B (Temp), AB, A^2^, B^2^, and AB^2^. The F-Value of this model was 17.63, indicating a significant model, with a 0.01% chance of an F-value of this size occurring due to noise. This model had a predicted R^2^ of 0.63 and an adjusted R^2^ of 0.75. To improve the model, three outliers, identified by using residuals vs. predicted plots, were omitted from the data (Run No.6, pH 3, Temp 4 °C, measured activity 20.62% and runs 26 and 27 pH 7, 37 °C, measured activities 22.62, 7.63 and 11.28%, respectively).

For human pepsin, A base 10 log transformation was used on the data due to a high maximum to minimum value ratio (100 to 0.6 = ratio of 165.4). Terms included in the model were the intercept, A (pH), B (Temp), AB, A^2^, B^2^, AB^2^, A^3^, and B^3^. The F-Value of this model was 29.39, indicating a significant model, with a 0.01% chance of an F-value of this size occurring due to noise. This model had a predicted R^2^ of 0.81 and an adjusted R^2^ of 0.86. To improve the model, one outlier, identified by using residuals vs. predicted plots, was omitted from the data (Run No.23, pH3, 37 °C – measured activity 14.3%).

The produced equations were integrated into separate Excel spreadsheets (Supplementary material Excel file), created to allow users to input specific pH and temperature values from their own *in vitro* digestions. These tools then allow to predict pepsin activity (either human or porcine) throughout digestion experiments as a percentage relative to the optimum conditions (pH 2 and 37 °C).

### Case studies

To test the spreadsheets developed and explore the potential uses of this work in future protein digestion studies, three case studies have been conducted.

**Case study 1** has been defined to test the model’s suitability to establish comparisons between projected levels of pepsin activity by human vs. porcine pepsin samples during gastric digestion *in vitro*. The conditions of an *in vitro* semi-dynamic gastric digestion of whole milk (20 mL), following standardised INFOGEST protocols, were used to capture pH and temperature data^11,20^.This data was input to the relevant pepsin activity prediction model, to predict the difference in activity between human and porcine pepsin.

**Case study 2** has been outlined to test the model’s usefulness in comparing predicted pepsin activity levels when using different *in vitro* digestion models. The conditions of a semi-dynamic vs. a static digestion protocol applied to whole milk were compared following the respective standardised INFOGEST protocols^11,20^.In both scenarios, the standard digestion temperature (37 °C) was considered and, in combination with the respective pH data, entered into the spreadsheets developed (see Supplementary Material) to estimate pepsin activity levels (expressed as the percentage of reported optimal porcine pepsin activity) in each scenario based on the predictive models that were developed in the present study.

**Case study 3** has been conducted to compare estimated pepsin activity levels in adults to those in older adults during digestion of a model meal, based on parameters extracted from the literature (Table 2). The basal gastric volume was set at 33 mL, as reported by Bryson Roberts, Sheers and Halstead Taylor (2007)^32^. The basal concentration of pepsin in the gastric fluid was 0.4 mg/mL, also referenced from Bryson Roberts et al. (2007)^32^. For the simulations, a meal volume of 50 mL containing 50 kcal was used. The gastric emptying rate was set to 2 kCal/min equalling 2 mL/min in this case^20^. The gastric secretion rate was established at 1 mL/min, set at normal fasting rate of secretion for the purpose of this model^33^. Additionally, for simulations modelling digestion in older adults (69–98 years old), a 40% reduction in pepsin output was incorporated, based on findings by Feldman, Cryer, McArthur, Huet and Lee^26^. These parameters were taken into consideration alongside gastric pH curves, based on approximate pH curves from two* in vivo* digestion studies to estimate total pepsin activity during an in vivo gastric digestion (Fig. 3C).

**Table 2:** Parameters selected to estimate total Pepsin activity *in vivo*.

Parameter	Value	Units	References
Basal gastric volume	33	mL	^33^
Basal pepsin concentration	0.4	mg/mL	^33^
Rate of pepsin increase in stomach	46.8	mg/hour	^33^
Meal volume	50	mL	-
Meal energy	50	kcal	-
Meal emptying rate	2	mL/min	^33,34^
Rate of gastric secretion	1	mL/min	
Pepsin output reduction in older adults	40	%	^26^

### Inactivation of pepsin

#### Impact of temperature and time

To determine the heat treatment required to irreversibly denature porcine pepsin, the following conditions have been tested. A porcine pepsin solution in water (20 µg/mL) was preheated to 65, 75, 85–95 °C for 5, 10–20 min. Following this, the pepsin was cooled to 37 °C and assayed under optimum conditions (pH 2, 37 °C) as described above. Three repetitions were carried out for each condition.

#### Study of conformational changes by fourier transform infrared spectroscopy

FTIR analysis was used to visualise structural changes in porcine pepsin activity following heat treatment at 65, 75, 85–95 °C for 5, 10–15 min.

A Bruker Invenio S with a temperature-controlled BioATR^®^ Zn/Secrystal (Bruker Optics, Ettlingen, Germany) was used, set at the above temperatures (65, 75, 85–95 °C). Liquid nitrogen was used to cool the MCT detector, and the sample compartment was purged to remove moisture and carbon dioxide. Bruker OPUS 5.5 software (Bruker, Germany) was used to obtain the spectra in absorbance mode. Each spectrum was measured in duplicate and consisted of 50 scans averaged. Spectra processing was carried out using the Bruker OPUS software in the form of vector normalisation and atmospheric correction (corrected for aqueous solutions, CO2 and water vapour). The background was measured with water and was subtracted from the spectrum. Spectra from native pepsin were subtracted from those of heat-treated samples^35^.

### Statistical analysis

Statistical analysis was conducted using Design Expert version 12.0 (Stat-Ease Inc., USA). The activities of human and porcine pepsin at the different temperature and pH combinations tested were fitted to a reduced cubic model, and analysis of variance (ANOVA) was used to evaluate the significance of the model and its terms. The quality of the model fit was assessed through the coefficients of determination: ^2^, adjusted ^2^, and predicted ^2^. Model adequacy was further verified using diagnostic plots, including the normal plot of residuals to evaluate the normality assumption and residuals versus predicted values to identify potential outliers and assess the homoscedasticity of residuals. Outliers were identified using residual vs. predicted plots in Design Expert version 12.0. Visualisation of data and results was performed using both Design Expert 12.0 and GraphPad Prism version 9.0 (GraphPad Software LLC, USA).

## Conclusion

This study demonstrates how human and porcine pepsin differ in activity across varying pH and temperature levels, showing the potential impact on proteolysis when substituting porcine pepsin for human pepsin in* in vitro* digestion protocols. Predictive models for pepsin activity were developed, providing tools to predict activity levels under different digestion scenarios and for various population groups.

A limitation of the models is their reliance on haemoglobin as a substrate, which may not fully reflect real food matrices. Future work should explore alternative substrates, such as caseins or plant proteins, to improve the applicability of these models. This study also confirms that a 5 min heat treatment at 75 °C is sufficient to irreversibly denature pepsin after digestion. These results contribute to a better understanding of* in vitro* gastric protein digestion and presents a tool for improving future digestion research.

## Supplementary Information

Below is the link to the electronic supplementary material.

Supplementary Material 1

Supplementary Material 2

## Data Availability

All data supporting the findings of this study are provided within the manuscript and its supplementary materials. Any additional data are available from the corresponding author upon reasonable request.
