# Supplementary material for: A tool for predicting pH and temperature effects on porcine and human pepsin activity during in vitro gastric digestion
Source: Sci Rep. 2026 Feb 15;16:9176. doi: 10.1038/s41598-026-38033-5 (PMC12996343; doi:10.1038/s41598-026-38033-5)
Supplement: Supplementary file 1 — Supplementary Material 1 [file 41598_2026_38033_MOESM1_ESM.docx]

Supplementary Material

***Supplementary Table 1: Human Gastric Fluid Pepsin Activity.***

| Run | A:ph | B:Temp (°C) | Pepsin Activity Human (% of maximum) | Pepsin Activity Porcine (% of maximum) |
| --- | --- | --- | --- | --- |
| 1 | 1 | 27 | 45.54 | 28.50 |
| 2 | 4 | 60 | 92.91 | 15.03 |
| 3 | 6 | 60 | 20.73 | 58.68 |
| 4 | 3 | 40 | 88.51 | 58.59 |
| 5 | 1 | 60 | 64.45 | 4.21 |
| 6 | 3 | 4 | ~~20.62~~ | 12.33 |
| 7 | 6 | 36 | 35.45 | 1.94 |
| 8 | 4 | 24 | 44.25 | *4.06* |
| 9 | 6 | 4 | 9.67 | *0.60* |
| 10 | *1* | *4* | *5.59* | *9.96* |
| 11 | 2 | 15 | 31.04 | *26.67* |
| 12 | 2 | 37 | 103.76 | 99.00 |
| 13 | *2* | *50* | *96.99* | 70.00 |
| 14 | 3 | 15 | 43.07 | 24.05 |
| 15 | 3 | 37 | 98.39 | 59.84 |
| 16 | 3 | 50 | 103.54 | 80.71 |
| 17 | 4 | 15 | 40.60 | *2.26* |
| 18 | 4 | 37 | 38.02 | *8.91* |
| 19 | 4 | 50 | 42.75 | 12.52 |
| 20 | 5 | 37 | 6.77 | 3.52 |
| 21 | 1 | 37 | 83.24 | 89.27 |
| 22 | 2 | 37 | 113.64 | 99.59 |
| 23 | 3 | 37 | 92.80 | ~~14.33~~ |
| 24 | *4* | *37* | *38.24* | 4.30 |
| 25 | 6 | 37 | 5.91 | 4.84 |
| 26 | 7 | 37 | ~~7.63~~ | 5.82 |
| 27 | 7 | 37 | ~~11.28~~ | 0.76 |
| 28 | 6.5 | 37 | 0.54 | 0.99 |
| 29 | 6 | 37 | 9.88 | 2.00 |
| 30 | 5.5 | 37 | 19.87 | 3.00 |
| 31 | 5 | 37 | 10.10 | 3.50 |
| 32 | 4.5 | 37 | 15.25 | 6.00 |
| 33 | 4 | 37 | 37.49 | *8.90* |
| 34 | 3.5 | 37 | 87.76 | 55.00 |
| 35 | 3 | 37 | 93.77 | 57.00 |
| 36 | 2.5 | 37 | 93.56 | 88.00 |
| 37 | 2 | 37 | 114.29 | 100.00 |

***Supplementary figure 1***

Supplementary Figure 1 Native Pepsin spectra (37 °C)
